# Efficacy of paliperidone palmitate 3-month formulation in preventing hospital admissions and emergency room visits. 66 months of follow-up

**DOI:** 10.1192/j.eurpsy.2023.1041

**Published:** 2023-07-19

**Authors:** S. L. Romero Guillena, B. O. Plasencia Garcia de Diego, J. Gomez Gonzalez, F. Gotor Sánchez-Luengo

**Affiliations:** 1 UGC Salud Mental Virgen Macarena, Psychiatry; 2 UGC Salud Mental Virgen Rocio, Psychiatry, Seville, Spain

## Abstract

**Introduction:**

Paliperidone Palmitate 3-month formulation (PP3M) has shown a significantly longer time to relapse compared to placebo, with similar efficacy and safety to Paliperidone Palmitate 1-month (PP1M) (Carpiniello et al. Drug Des. Devel. Ther. 2016; 10 1731–1742).

**Objectives:**

The main objective of this study was to determine the effectiveness of PP3M in preventing hospital admissions and emergency room visits, in people with non-acute schizophrenia in a naturalistic psychiatric outpatient setting

**Methods:**

Sample: 30 people with diagnosis of schizophrenia (DSM 5 criteria), who had started treatment with PP3M, after being stabilized with PP1M (the dose was not modified in the four months prior to inclusion in the study)

Quarterly basis, the following evaluations were performed during a follow-up period of 66 months:

The Clinical Global Impression-Schizophrenia scale (CGI-SCH)

Treatment adherence, concomitant medication and the number of hospitalizations and emergency visits

Efficacy values: Percentage of patients who remained free of admissions at the end of 66 months of follow-up.

Other evaluation criteria: Percentage of patients who never visited the emergency department at the end of 66 months of follow-up. Average change from baseline visit to the final evaluation as assessed by score obtained on the following scale: GSI-SCH, percentage of patients on antipsychotic monotherapy and treatment adherence rate.

**Results:**

The mean dose of PP3M was 401. 55 mg

The percentage of patients who remained free of admissions at the end of the 66 months was 83.25% and the percentage of patients who never visited the emergency department at the end of 66 months was 79.92%

Mean variations from baseline scores at 66 months were: (-0.36 ±0-37) on the GCI-SCH.

The percentage of patients on antipsychotic monotherapy at the end of the 66 months was 76.56%

The rate of adherence was 86.58%

.

**Conclusions:**

In our study, we found that paliperidone palmitate 3-month formulation was effective in reducing the number of admissions and visits to the emergency department, under conditions of daily clinical practice.

**Disclosure of Interest:**

None Declared

